# Study protocol for a cluster randomized controlled trial to test “¡*Míranos*! Look at Us, We Are Healthy!” – an early childhood obesity prevention program

**DOI:** 10.1186/s12887-019-1541-4

**Published:** 2019-06-10

**Authors:** Zenong Yin, Sarah L. Ullevig, Erica Sosa, Yuanyuan Liang, Todd Olmstead, Jeffrey T. Howard, Vanessa L. Errisuriz, Vanessa M. Estrada, Cristina E. Martinez, Meizi He, Sharon Small, Cindy Schoenmakers, Deborah Parra-Medina

**Affiliations:** 1 Department of Kinesiology, Health and Nutrition, The University of Texas at San Antonio, San Antonio, TX USA; 2 Department of Epidemiology and Public Health, The University of Maryland, School of Medicine, Baltimore, MD USA; 3 Department of Mexican American and Latina/o Studies Austin, The University of Texas at Austin, Austin, TX USA; 4 Parent/Child Incorporated of San Antonio and Bexar County, San Antonio, TX USA; 5 Family Service Association of San Antonio, Inc., San Antonio, TX USA

**Keywords:** Obesity, Preschool children, Policy, Physical activity, Sedentary time, Nutrition, Sleep, Parent, Home, Childcare

## Abstract

**Background:**

One in three Head Start children is either overweight or obese. We will test the efficacy of an early childhood obesity prevention program, “¡*Míranos*! Look at Us, We Are Healthy!” (¡*Míranos*!), which promotes healthy growth and targets multiple energy balance-related behaviors in predominantly Latino children in Head Start. The *¡Míranos!* intervention includes center-based (policy changes, staff development, gross motor program, and nutrition education) and home-based (parent engagement/education and home visits) interventions to address key enablers and barriers in obesity prevention in childcare. In partnership with Head Start, we have demonstrated the feasibility and acceptability of the proposed interventions to influence energy balance-related behaviors favorably in Head Start children.

**Methods:**

Using a three-arm cluster randomized controlled design, 12 Head Start centers will be randomly assigned in equal number to one of three conditions: 1) a combined center- and home-based intervention, 2) center-based intervention only, or 3) comparison. The interventions will be delivered by trained Head Start staff during the academic year. A total of 444 3-year-old children (52% females; *n* = 37 per center at baseline) in two cohorts will be enrolled in the study and followed prospectively 1 year post-intervention. Data collection will be conducted at baseline, immediately post-intervention, and at the one-year follow-up and will include height, weight, physical activity (PA) and sedentary behaviors, sleep duration and screen time, gross motor development, dietary intake and food and activity preferences. Information on family background, parental weight, PA- and nutrition-related practices and behaviors, PA and nutrition policy and environment at center and home, intervention program costs, and treatment fidelity will also be collected.

**Discussion:**

With endorsement and collaboration of two local Head Start administrators, ¡*Míranos*!, as a culturally tailored obesity prevention program, is poised to provide evidence of efficacy and cost-effectiveness of a policy and environmental approach to prevent early onset of obesity in low-income Latino preschool children. *¡Míranos!* can be disseminated to various organized childcare settings, as it is built on the Head Start program and its infrastructure, which set a gold standard for early childhood education, as well as current PA and nutrition recommendations for preschool children.

**Trial registration:**

ClinicalTrials.Gov (NCT03590834) July 18, 2018.

**Electronic supplementary material:**

The online version of this article (10.1186/s12887-019-1541-4) contains supplementary material, which is available to authorized users.

## Background

### Childhood obesity and energy balance-related behaviors

Childhood obesity is a complex, multifactorial health problem that extends into adolescence and adulthood and leads to increased cardiometabolic risks [1, 2], as well as psychosocial and economic burdens [3, 4]. While the epidemic of obesity remains apparent in preschool children in the United States (U.S.) [5], young children aged 3–5 from certain racial/ethnic groups and from low-income families are disproportionally affected [6–8]. For example, the prevalence of obesity in Hispanic children aged 3–4 enrolled in the Special Supplemental Nutrition Program for Women, Infants, and Children (WIC) was 19.1% in New York City and 21.7% in Los Angeles County in 2011 [9]. Obesity (accumulation of excessive adipose tissue) results from the imbalance of energy intake and expenditure and the dysregulation of energy balance-related behaviors (EBRBs) [10, 11]. For preschool children, primary EBRBs include dietary behaviors [12, 13],moderate to vigorous physical activity (MVPA) [14], sedentary behavior [15], and sleep [16]. Latino children possess higher numbers of risk factors for obesity and dysregulation of EBRBs than non-Latino children [7, 17].

It is recommended that preschool children should engage in ≥90 min (min) of MVPA daily [18], including 60 min of structured play and up to several hours of unstructured play, and should not be sedentary for more than 15 min at a time [19]. However, these recommendations are not widely endorsed and/or implemented by childcare providers [20]. Furthermore, obese children are less active [21] and have lower levels of gross motor skills [22] compared to their normal-weight peers. A meta-analysis of 29 studies of preschoolers aged 3–5 found that the average MVPA was 42.8 min/day(d) [23], while a 2012 review of five prospective studies linked watching TV > 2 hr/d with increased body mass index (BMI) and skinfolds after controlling for PA in preschool children [15]. Alarmingly, U.S. preschool children spend 73–84% of their waking hours sedentary [24]. Recently, insufficient sleep (≤11 h/d) was linked with increased risk for obesity in preschool children [25, 26]. In a large cohort of U.S. children aged 3–12, those sleeping ≥11 h/d at baseline had a 26% lower risk for being overweight compared to those sleeping 9–10 h/d at the 5-year(y) follow-up [27]. The study also found each additional hour of sleep was associated with a reduction of BMI by .12 standard deviation [27]. Not surprisingly, TV watching leads to insufficient sleep in children [28]. Therefore, effective strategies for promoting MVPA and gross motor skills, reducing sedentary behavior and promoting adequate sleep are critical for obesity prevention in preschool children [29].

Available data reveals that American preschool children do not consume a balanced, healthy diet [30]. According to a cross-sectional analysis of 2005–2010 National Health and Nutrition Examination Survey (NHANES), children of all ages scored far below the minimum federal guideline for good health based on a Health Eating Index-2000 score [31]. A separate analysis using 2-d dietary recalls of 2007–2010 NHANES found that only 0.01 to 29% of children ≤8 y old met the sex- and age-based food group recommendations for total vegetables, whole grains, refined grains, and energy intake from solid fats and added sugars [32].

Food environment critically influences the formation of eating preferences and habits during preschool years [33, 34]. Modifications of parent feeding practices such as offering healthier foods or reducing energy-dense food can increase the intake of nutritious food and lower total energy intake in preschoolers [34]. Others showed that consuming sugary drinks was found to be associated with obesity [35], while serving water and limiting sugary drinks may reduce obesity in preschoolers [13]. Strategies addressing these dietary practices can reduce excessive energy intake [36] without interfering with children’s ability to self-regulate their energy intake [37].

An integrated approach is urgently needed to combat childhood obesity by addressing key enablers and barriers [38] that influence children’s EBRBs [3, 39]. An emerging consensus points to four key enablers and/or barriers for the successful prevention of obesity in children attending organized childcare: 1) physical activity (PA) and nutrition policy and environment; 2) staff development and training; 3) parental practices/family engagement; and 4) cultural tailoring of intervention delivery [20, 40]. Because 60% of preschool children in the U.S. attend organized care [41], a multi-level, multi-setting approach to address these key enablers and/or barriers holds great promise to prevent obesity in this age group [42].

### Development of ¡*Míranos*! Look at us, we are healthy! (¡*Míranos*!)

In collaboration with local Head Start administrators, a multi-disciplinary research team developed and pilot-tested ¡*Míranos*!, a culturally tailored obesity prevention program to address the needs and challenges facing low-income, predominantly Latino preschool children [43–45]. Head Start is a federal program that provides school readiness and support services (e.g., health, nutrition, social services) to low-income children aged birth to 5 and their families [46]. Alarmingly, one in three Head Start children is overweight or obese [47], a much higher ratio than the national average. Because Head Start focuses on children’s cognitive and social development as well as health, mandates parent involvement [48], and proactively promotes PA and healthy eating [49, 50], obesity prevention in this vulnerable population has great potential for long-term impact [20]. Our overarching goal is to take advantage of the synergy of changes at different levels of influence and in multiple settings [38] to increase the likelihood of developing long-term health habits that reduce daily energy imbalance gaps [51] by targeting multiple EBRBs in the childcare setting and at home.

Working with Head Start administrators, staff and parents, we identified two approaches: the Center-Based Intervention (CBI) focusing on modifying the policies, practices, and environment in Head Start centers and the Home-Based Intervention (HBI) targeting parental health practices and the home environment. We used intervention mapping to identify and develop strategies from evidence-based guidelines and recommendations and published studies to target enablers/barriers in childcare and home environments [52].

**Fig. 1:**
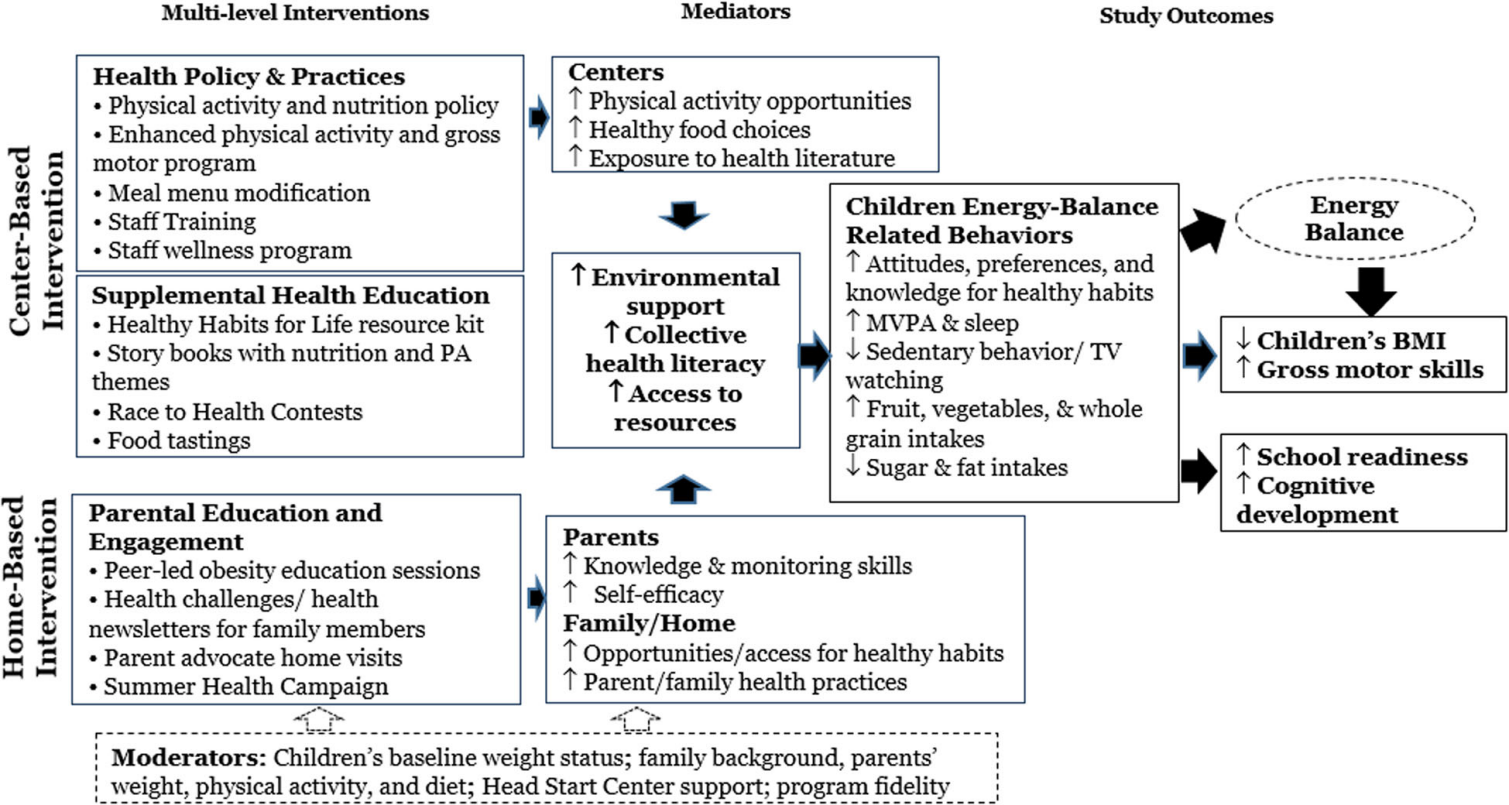
*¡Míranos!* Intervention conceptual model

We conducted a series of pilot studies to develop and refine the ¡*Míranos*! intervention program. The design of the intervention was guided by a systems perspective to: 1) map strategies that address the enablers and/or barriers of obesity prevention in Head Start [53, 54]; 2) coordinate a multilevel effort [38] that will target multiple EBRBs [33, 55]; 3) identify mediators and moderators between settings and study outcomes [54]; and 4) address cultural relevance [56]. In developing interventions, we utilized: 1) theories of early childhood development to provide children with cognitively and developmentally appropriate activities;^135^ 2) social cognitive theory to increase behavioral knowledge and skills and self-efficacy with direct learning, role-modeling and reinforcement in Head Start staff and parents [57]; and 3) a socioecological model to conceptualize interventions at the individual, family, organizational and policy levels [58]. Key components of these theories applied to the ¡*Míranos*! intervention are presented in the conceptual model depicted in Fig. 1.

## Methods and design

### Design and study aims

The study will use a cluster randomized controlled design to test the efficacy of the ¡*Míranos*! intervention in preventing excessive weight gain and promoting the development of healthy habits in young children enrolled in Head Start. The primary end point for the study is a change in BMI at the posttest (7 mo from baseline). Using a three-arm design, 12 Head Start centers will be randomly assigned to one of three conditions in equal number: 1) a combined center- and home-based intervention, 2) center-based intervention only, or 3) comparison. The interventions will be delivered by trained Head Staff staff during the academic year. A total of 444 3-year-old children (*n* = 37 per center) will be enrolled in the study in two cohorts at baseline and followed prospectively 1 year post intervention. The first cohort will be recruited between May 2018 and September 2018. The second cohort will be recruited between May 2019 and September 2019. Outcome assessment will be conducted at baseline (T0), immediate post-intervention (T1), and at the one-year post-intervention follow-up (T2; 21 mo from baseline). The assessment at each time point will take up to 4 days to complete at a center, depending on the size of the center enrollment. We will divide the 12 centers into four groups to assure the manageability of data collection and the intervention delivery. Each group includes a center from each condition to control the extraneous conditions (e.g., weather conditions, organizational events) and secular trends. The intervention will commence in the week following the completion of the assessment.

Specific aims and hypotheses of the study are to:

Aim 1: Test the efficacy of the *¡Míranos!* intervention on healthy weight growth measured by BMI change (primary outcome) in normal weight, overweight and obese children. Hypothesis 1: Children in the combined center- and home-based or center-based intervention conditions will have a significantly smaller increase in BMI (kg/m^2^) compared to children in the comparison condition at T1 and T2.

Aim 2: Test the impact of the *¡Míranos!* intervention on children’s PA and dietary behaviors (secondary outcomes). Hypothesis 2: Children in the combined center- and home-based or center-based intervention condition will have significantly higher levels of MVPA, gross motor skills, sleep duration and intakes of fruits, vegetables, and whole grains, as well as lower levels of sedentary behavior, TV watching, and intake of sugar/fructose and fat, compared to children in the comparison condition at T1 and T2.

Aim 3: Evaluate the cost-effectiveness (CE) of the *¡Míranos!* intervention. Standard trial-based CE analysis methods will be used to estimate net intervention costs per unit of BMI reduction in each of the treatment groups compared to the control group, from the program provider perspective. Information on the CE of different intervention approaches will help the decision maker (provider/payer of the program) maximize population health subject to the available resources. This critical information is missing in the current literature.

### Study setting, recruitment, and randomization

Two Head Start administrators in San Antonio, Bexar County, Texas have joined the study as collaborators and agreed to randomize their centers as the study sites. Both organizations have previously worked with the study team in developing and piloting the intervention program. The two organizations represented by these administrators oversee 49 Head Start centers with a total enrollment over 2000 children. According to the published eligibility criteria of the Administration for Children and Families of the U.S. Department of Health & Human Services, “children from birth to age five who are from families with incomes below the poverty guidelines are eligible for Head Start and Early Head Start services.” Children from homeless and foster families and families receiving other forms of public assistance are also eligible. The study eligibility criteria for the centers and children are displayed in Table 1. Children will not be excluded from the study if they do not speak English or have limited English proficiency. After discussing the eligibility issues with the two Head Start organizations, the research team determined that three centers from organization A and nine centers from organization B that meet the center inclusion eligibility criteria will become the study sites. Using statistical software R (version 3.3.2), the centers are randomly assigned to one of the three treatment conditions stratified by the organizations and the center enrollment size so that the centers from both organizations are equally represented in the study.

**Table 1:** Study eligibility criteria

Center eligibility	1. A full-day center (offering ≥7 h of care/day)
	2. At least one classroom enrolling children aged 3
	3. Agreement to modify center physical activity and nutrition policies
	4. Agree not to participate in other health-related studies.
Child eligibility	1. Enrollment in a study center
	2. Age 3 years at baseline
	3. One child per family
	4. Parental consent

The recruitment of child participants (participant recruitment) will take place during the registration period in the summer and before the baseline assessment in September by sending a recruitment packet to child’s home. The content of the packet includes: *¡Míranos!* study information sheet, recruitment flyer, informed consent form, and a letter from center director and study PIs. Parents/guardians (parents) may either 1) review the information about the *¡Míranos!* study, complete the informed consent form, and return the signed consent form to the center director in a sealed envelope, or 2) take the packets home and mail the signed consent form in a prepaid envelop to the UTSA research team. Parents will be provided with a phone number to call the study team if they have questions. Children will receive a coloring book if their parents return a signed consent form either agreeing or declining to participate in the study.

### Intervention and control condition

The design of the *¡Míranos!* intervention focuses on key messages that promote the development of healthy habits in young children. These key messages are based on available evidence that target the EBRBs to promote energy balance and reduce the risk of obesity. These key messages are displayed in Table 2. All intervention activities are reflective of these key messages.

**Table 2:** ¡Míranos! Intervention Key Messages

PA and Nutrition Policies	1. Educate children to develop healthy habits for life
	2. Offer 90-min free, teacher-led physical activity to children at the center everyday
	3. Offer balanced healthy meals and snacks utilizing the USDA Child and Adult Care Food Program best practice recommendations
Staff	1. Be part of children’s play
	2. Role-model healthy behaviors to children at all times
	3. Be physically active 30 min everyday
	4. Eat healthy MyPlate meals everyday
Parents	1. Help your child get 30 to 60 min physical activity at home everyday
	2. Serve fruits and vegetables to your child at every meal
	3. Limit your child’s TV watching to less than 2 h everyday
	4. Avoid offering sugar-added beverages to your child
	5. Turn TV off during meals
	6. Help your child get at least 10 h of sleep everyday

#### ¡Míranos! Center-based intervention

CBI has four components that are designed to enhance the support and opportunities for increasing PA, reducing sedentary time, and promoting healthy eating.

##### PA and nutrition policy and environment

Center policy and environment are modified based on the current evidence-based recommendations and guidelines and represent significant changes to the ongoing practices in Head Start. Both Head Start organizations have endorsed the proposed modifications and will require the center directors to create a daily schedule and change daily routines to facilitate the implementation of the policy changes at all intervention centers. To increase centers’ compliance, the central office curriculum staff have collaborated with the research team to develop written policies and guidelines and to provide training and technical assistance on new policy and practices. The Head Start program follows the meal pattern guidelines of the Child and Adult Care Food Program (CACFP) of the U.S. Department of Agriculture, which is based on the Dietary Guidelines for Americans. The research team has worked with food services staff from center kitchens to incorporate the optional best practice recommendations from CACFP that will further improve the nutritional quality of the meals. These best practices include an increase in the serving frequency of fresh fruit, vegetables, and whole grain foods and a reduction in the serving of sugar and fats. Meal modifications for the intervention centers are covered by supplemental funding from the study. Specifically, meal pattern modification includes 1) serving fruit and vegetables at snacks [2–3 times/week]; 2) adding one serving of a dark leafy green, one of an orange/red fresh vegetable, and one legume/bean serving per week; and 3) utilizing more seasonal fruits and vegetables. To assure the success of implementation, the research team and both Head Start organizations have signed a Memorandum of Understanding to confirm their support for and participation in the study. Tables 3 and 4 show the physical activity and nutrition policies that will be implemented in the intervention centers. The policy modifications are modeled following “Model Policies for Creating a Healthy Nutrition and Physical Activity Environment in Child Care Settings” developed by the Missouri Department of Health and Senior Services, Bureau of Community Food and Nutrition Assistance. Table 5 displays the expecatations and goals for delivering the center-based intervention activities.

**Table 3:** Physical Activity Policies

Policy Area: Active Play and Inactive Time	
Policy #1	Children will have at least of 90 min of structured and unstructured playtime each school day.
Policy #2	Children will participate in outdoor active play two times or more each school day.
Policy #3	Children will participate in morning outdoor play (structured activity 15 min and free play 15 min) each school day.
Policy #4	Children will participate in active learning classroom activities during center time, transition, and breaks (30 min) each school day.
Policy #5	Children will participate in afternoon outdoor play (structured activity 15 min and free play 15 min) each school day.
Policy #6	Screen time for entertainment at the center will be limited to 30 min per week.
Policy #7	Children’s sitting time will be < 15 min in any setting except nap and meal time.
Policy Area: Play Environment	
Policy #8	Each child will have a piece of play equipment during structured play.
Policy #9	A variety of portable play equipment will be available for children to use at the same time during free play.
Policy #10	Heat Start teachers and teaching aids will lead and participate in physical activity with children.
Policy #11	Play area will be safe for children to play.
Policy Area: Supporting Physical Activity	
Policy #12	Head Start staff will encourage children to engage in active play without pressure.
Policy #13	Head Start staff will not withhold playtime as punishment for children’s misbehaviors.
Policy #14	All Head Start center staff will complete a mandatory, paid training on obesity prevention, physical activity and nutrition.

**Table 4:** Nutrition Policies

Policy Area: Mealtime Environment	
Policy #1	New fruits and vegetables will be introduced through structured food tastings. Non-food rewards will be given for participation.
Policy #2	Children will never be forced to eat or try new foods. Children will decide how much to eat at every meal and snack.
Policy #3	Food will not be given as a reward or taken away as punishment.
Policy #4	Staff members will sit at the table with children during meals and snacks.
Policy #5	Staff members will model healthy behavior by consuming the same food and drinks as the children and will not consume other foods and drinks in front of the children.
Policy #6	Meals will be served family style.
Policy Area: Nutrition Education	
Policy #7	Teachers will incorporate Healthy Habits for Life into current curriculum and deliver lessons to children.
Policy #8	Staff will have the opportunity to participate in a free staff wellness program.
Policy #9	Healthy contests coordinated with the Healthy Habits for Life curriculum and staff wellness program will encourage children and staff to participate in healthy behaviors. Non-food rewards will be given for student and staff participation.
Policy Area: Foods from Outside the Facility	
Policy #10	The center will have guidelines for foods or nonfood items brought into the facility and served for holidays and celebrations.
Policy #11	Holidays will be celebrated with mostly healthy foods and nonfood treats.

**Table 5:** Expectations and Goals for Delivering the Center-based Intervention Activities

Outdoor play sessions (morning and afternoon):	1. 60 min of physical activities
	a. 15-min teacher led activities using Miranos! Activity Cards
	b. 15-min free play
	c. Join the children in play
	d. Have play equipment out for free play
Health education activities from Healthy Habits for Life:	1. Read/sing HHL poem at the beginning of the day
	2. Display HHL “Did You Know” poster at entrance for parents to read
	3. Teach each HHL activity at least 2 times a week
	4. Watch The Get Healthy Now Show 2–3 times a week (5–10 min at a time; do not watch the whole show in one setting)
	5. Read Miranos! storybook for the week at least twice a week
	6. Install YouTube version of all Miranos! storybooks on Learning Center computers
Transition activities that will keep children physically active:	1. 15 min of physical activities
	a. Using GoNoodle
	b. Using music on tablet
	c. Active learning activities during Learn Centers
	d. Goal: 15 min of physical activities
	e. Not sitting longer than 15 min
	f. Use Learning Ladder
	g. Use Miranos! activity cards
Health contest:	1. Track each child’s participation daily
	2. Post the contest results
Staff wellness:	1. Complete weekly activity for each week
	2. Participate in health challenges
Evaluation survey:	1. Complete the evaluation survey of all Miranos! activities by Friday

##### *¡Míranos!* PA/gross motor program

Head Start children will participate in daily PA (30-min structured and 60-min non-structured play) during outdoor/indoor play, learning center time, and transitions. Teachers will use *¡Míranos!* Activity Cards (at least one card/day) and equipment supplied by the study to meet the PA goals (see Additional file 1 for samples of Activity Cards). The Activity Cards are written lesson plans to increase MVPA and teach age-appropriate gross motor skills in structured and unstructured group formats for outdoor and indoor settings. The Activity Cards were designed by physical education specialists according to principles of motor development and can also be used during transitions and learning centers. Portions of the Activity Cards are written based on the storylines of 21 children’s books with nutrition and PA themes that can be readily integrated into daily routine activities (e.g., story time, transition). We also created active learning activities (e.g., learning ladders) that combine literacy and numeracy skills with physical activities, which can be used by the learning centers to increase opportunities for PA. Teachers will also use age-appropriate movement music CDs and dance videos that can be used for brain break activities after 15-min sedentary time and provide PA alternatives for indoors and bad weather days. The teachers will identify and include the structured and non-structured activities into their daily lesson plans. We will develop a training DVD to detail lesson implementation and demonstrate gross motor activities to help teachers develop confidence and overcome challenges in leading the activities and to reinforce key concepts from the staff training.

##### Supplemental health education activities

The Sesame Workshop bilingual Healthy Habits for Life (HHL) resource kit is the primary source for health education. The HHL uses Sesame Street cartoon characters to promote PA and healthy eating in children aged 3–5. There are 9 modules with short, age-appropriate learning activities, hands-on games, and interactive DVD activities (The Get Healthy Now Show) that can be integrated into daily center routines. Each module has a “Did You Know” fact to promote a key health message to children and parents. At least one storybook will be introduced during story time that is related to the weekly topic of the HHL. Head Start teachers will incorporate HHL activities into their daily lesson plans with a goal of using all activities in each module at least once a week. Health contests will be conducted to increase PA and intake of water, fruit, vegetables, and reduce TV watching and sugar-added drinks in accordance with HHL topics. “Did You Know” facts will be displayed with signboards at center entrance and classrooms to promote evidence-based health messages to children and parents.

The Head Start center directors and teachers will integrate PA and nutrition education activities into daily lesson and routines following the *¡Míranos!* master intervention schedule during the biweekly lesson planning required by Head Start standards of practice. The *¡Míranos!* master intervention schedule shows the coordination and outlines the weekly activities for each component of the CBI. The teachers will submit their lesson plans to the center director for review and feedback. Table 4 shows the expectations and goals for Head Start teachers to deliver the center-based activities. To facilitate the integration of the intervention activities by Head Start teachers, we created a *¡Míranos!* eBook that provides weekly intervention schedules, electronic copies of intervention activities, access to online movement music and videos, and online audio/video versions of the children’s storybooks. Each teacher and center director can access the content of the eBook on an Android tablet. Each intervention classroom is equipped with a Smart TV monitor that can be linked to the eBook to display the eBook content (e.g., HHL poems, HHL video, electronic storybooks) and to show GoNoodle videos and other music videos to the children in the classroom for bad weather days and for transition activities.

#### ¡Míranos! Staff wellness program

A staff wellness program, which consists of a staff wellness manual and challenges, was developed to align with the *¡Míranos!* curriculum with the goal of encouraging staff to improve their own health and become healthy role models for the children at the center. The staff wellness manual, created based on information provided by the US Dietary Guidelines for Americans 2015 and the Centers for Disease Control and Prevention, utilizes Knowles’ Principles of Andragogy to establish topic relevance and social cognitive theory to enhance self-efficacy through goal-setting. The manual contains three main sections: 1) physical activity and hydration; 2) fruits and vegetables; and 3) overall wellbeing. Each section provides benefits for each health behavior, evidenced-based recommendations, examples and tips, suggested exercises or recipes, and goal-setting worksheets. Detailed instructions for use are included along with a weekly calendar that assigns staff wellness manual sections and staff wellness challenges to the *¡Míranos!* content at each site. Center-wide staff challenges, initiated by the center director, coincide with the children’s health contests. Each center director will post flyers 1 week prior and during the staff challenge to encourage participation. Posters to track staff progress will be posted in a staff-only area and center directors will report the number of staff who participated in the challenge and who achieved their goal to receive cash incentives for their center. Participation in the staff wellness program is voluntary and coordinated by the center directors.

#### Home-based intervention

The home-based intervention (HBI) arm of the *¡Míranos!* study is designed to engage parents/guardians of Head Start children and to educate them on child obesity prevention. Centers assigned to the HBI will provide parent education through several components, including peer-led obesity education, newsletters, family health challenges, and home visits with Head Start staff. The HBI consists of eight peer-led parent education sessions with take-home activities, eight family health challenges, sixteen parent newsletters, summer resource packet, and three home visits.

##### Peer-led obesity education

Head Start requires parents/guardians to physically sign their child in and out of the center. Seizing on this opportunity to engage parents, trained Head Start parents will deliver eight monthly peer-led education sessions using wall posters, live demonstrations, and instant feedback during child pick-up time. A wall poster session can be completed in 15–20 min. During the education sessions, six posters will be used to highlight parental beliefs and practices and to teach current guidelines and recommendations for child PA and nutrition. Use of posters in education sessions also allows peer educators to promote evidence-based strategies related to positive child feeding, increasing PA and sleep duration, reducing screen time at home, and limiting sugary drinks and promoting water. Session topics and activities are displayed in Table 6. All wall posters will be bilingual.

**Table 6:** Parent education poster session topics

Session #	Topic
1	Overview of *¡Míranos!*
2	Physical Activity Benefits and Recommendations
3	Limiting Screen Time at Home
4	Balanced Diet and Expert Recommendations
5	Keeping Healthy Foods in the Home
6	Promoting a Balanced Diet and Healthy Eating Habits
7	Promoting Physical Activity Indoors and Outdoors
8	Sleep, Bedtime Routine, and Expert Recommendations

##### Peer educator training

The Head Start Center Director/Operator at each center will identify and recruit four to six parents from their center to serve as peer educators and deliver the sessions. Qualifications include speaking English and Spanish and a history of volunteering at a center. Peer educators will receive a small stipend (up to $240) to participate in multiple trainings and deliver the sessions, for a total of 32 h of work across 8 months.

##### Take-home bag

During peer-led education sessions, parents will be asked to complete a scavenger hunt, a sheet of paper with six questions that pertain to the session topic (e.g., True or False? Experts recommend that preschoolers get at least 2 h a day of physical activity). Answers to the scavenger hunt questions are found by visiting the posters and interacting with peer educators. Parents who complete the scavenger hunt will receive a take-home bag that includes a health-themed storybook, a bilingual, family activities newsletter, and a developmentally-appropriate interactive game.

##### Family newsletter

As part of the HBI, 16 biweekly, bilingual *¡Míranos!* Health Newsletters will be sent home in take-home bags at the end of each education session (*n* = 8 newsletters) and in the child’s daily home folder (*n* = 8 newsletters). These newsletters, designed for 5th-grade reading comprehension, will provide information and tips for parents/guardians to help modify their family’s health behaviors related to physical activity, diet, screen time, and sleep so that they can support and role-model to their child. Additionally, each newsletter provided in the take-home bag will provide a culturally appropriate healthy snack or meal recipe that parents can easily make at home, as well as a low-cost or free community resource (e.g., a city park or event) that parents can attend with their children to help promote a healthier lifestyle.

##### Family health challenge

Immediately following each peer-led education session, parents will receive a “Family Health Challenge” form in their child’s take-home folder that involves the whole family on a targeted health behavior (e.g., drinking water, limiting screen time, and increasing physical activity) that relates to the topic of the education session. Parents will be able to choose from one of three challenges for their family to complete over the course of 7 days. Parents will mark on the form whether or not they completed the challenge. Children whose parents have returned a completed health challenge form will have their names publically displayed in a poster in the classroom.

##### Home visits

Per Head Start standards, Head Start Family Service Workers who have training in social work conduct two home visits per year at a minimum (~ 30 min/visit), and additional visits if needed. During the visits, the Family Service Workers will identify needs and issues, devise an improvement plan, if needed, and provide monitoring and support to parents. We will integrate a protocol into three home visits to develop skills and strategies for parents to promote PA, nutrition, screen time, and sleep at home. Each home visit will have two different health topics that Family Service Workers will introduce to the parent. Home Visit 1 will focus on increasing physical activity and limiting screen time, Home Visit 2 on increasing fruit and vegetable intake and limiting sugary drinks, and Home Visit 3 on healthy eating practices and sleep. As part (~ 15 min) of each home visit, the Family Service Worker will review the two health topics with the parent by providing an informational handout. The parent will then choose one of the health topics to set a family goal and develop a *¡Míranos!* Action Plan (a log for parents to document their participation and progress per the Head Start requirement). The Family Service Worker will guide the parents to establish family rules and develop strategies from a menu of evidence-based strategies to achieve their goal and make the home environment more conducive for healthy behaviors. For example, to implement the rule of sleeping ≥10.5 h/d, parents can remove TVs from children’s bedrooms and establish bedtime routines. The Family Service Worker will record the parents’ chosen rules and strategies in the *¡Míranos!* Action Plan and follow-up with parents after 1 month to track progress. At Home Visits 1 and 3, the Family Service Worker will ask parents to complete the Home Environment Questionnaire to identify the availability and accessibility of healthy and unhealthy foods in the home, electronics and play equipment in the home, and child sleep duration and bedtime routines. This will allow research staff to determine whether home visits impacted the home environment*.*

#### Staff development and training

We will provide development training 1) to increase Head Start staff health literacy (e.g., knowledge in obesity, nutrition, and PA), and instruction and management skills (e.g., role-modeling, PA skill demonstration, and leading activities, positive reinforcement), and 2) to implement ¡*Míranos!* intervention activities. All Head Start staff, including teachers, teaching assistants, Family Service Workers, center directors, food service workers, and custodians, will complete a paid training of up to 20 h depending on the roles of the staff in the study. The training includes online didactic education modules on physical activity and nutrition (8 h, required for all staff) and two half-day in-person training sessions (4–12 h, required depending on roles in the study). The in-person training is designed to familiarize the staff with the ¡*Míranos!* intervention protocol and the physical activity and nutrition policy modifications. Training topics include the study overview and protocol (1.5 h); center policy modifications (1 h); intervention program components (1.5–2.5 h); intervention coordination (30 min); physical activity and gross motor skill instruction (50 min); demonstration of *¡Míranos!* gross motor and physical activities for outdoor and indoor settings, transition and active learning activities, and use of equipment (2 h); health/nutrition education and instruction (50 min); demonstration of *¡Míranos!* health education activities, equipment, and supplies (40 min); and administrative issues (1 h).

Family Service Workers will receive separate training to implement the HBI. Each month, the peer educators will attend a training (1.5*)* with the Head Start Education Specialist (ES) or Education Center Coordinator (ECC) assigned to their center to prepare for parent education sessions. During trainings, peer educators will review information about the topic for the session (i.e., Physical Activity Recommendations and Benefits, Balanced Diet Recommendations, Keeping Healthy Foods in the Home, etc.) by watching a short (10–15 min) video, developed by research staff, that leads peer educators through the information displayed on each of the 6 posters for that session. Peer educators will also receive poster scripts, documents that contain key information for each of the posters that peer educators should relay to parents attending the education sessions. The poster scripts reflect the information presented in the training videos and are supplements that the peer educators will use during education sessions. Peer educators will actively practice the session content by role-playing during each training. Peer educators will pair up and take turns playing the role of center parent while the other practices poster content. At the end of each training, ES/ECC will prompt peer educators to discuss anticipated or experienced challenges during education sessions and problem-solve to address any identified challenges for the next education session.

Booster trainings (5 h) will be conducted to provide additional training based on needs during the year. Additional training will be provided at a later time to those who did not complete the initial training. All peer educators will receive a certificate upon completing the training. We will also develop the training of the Family Service Workers in implementing the home visits, including conducting home audits, developing the *¡Míranos!* Action Plan, and counseling/problem solving. We will make a DVD of training modules for staff, Family Service Workers, and peer educators that can be used later to train new staff.

#### Comparison condition

The study Head Start organizations have adopted “I Am Moving, I Am Learning” (IMIL) as its required PA and nutrition curriculum since FY 2012. IMIL is an obesity prevention program developed for and endorsed by Head Start for increasing the time in MVPA and structured PA and encouraging children to take healthy food choices by educating Head Start staff and parents. Head Start directors and staff can design their own program using activities and materials (games and gross motor activities, one set of play equipment, nutrition activities, and parent newsletters) from an IMIL kit after a brief training by an IMIL facilitator. The control centers will continue using IMIL. Although a classic “no treatment” control is common in RCTs, our study participants and partners are more receptive to a control condition that offers some attention and benefits. All comparison children will get some education on PA and nutrition via IMIL; in addition, we will deliver a literacy education program to children in comparison centers to increase buy-in and retention. The literacy education program, called Book Bites sponsored by a local grocery chain, will include 30-min sessions that incorporate early childhood literacy and nutritional concepts through interactive book reading activities.

### Trial flow

**Fig. 2:**
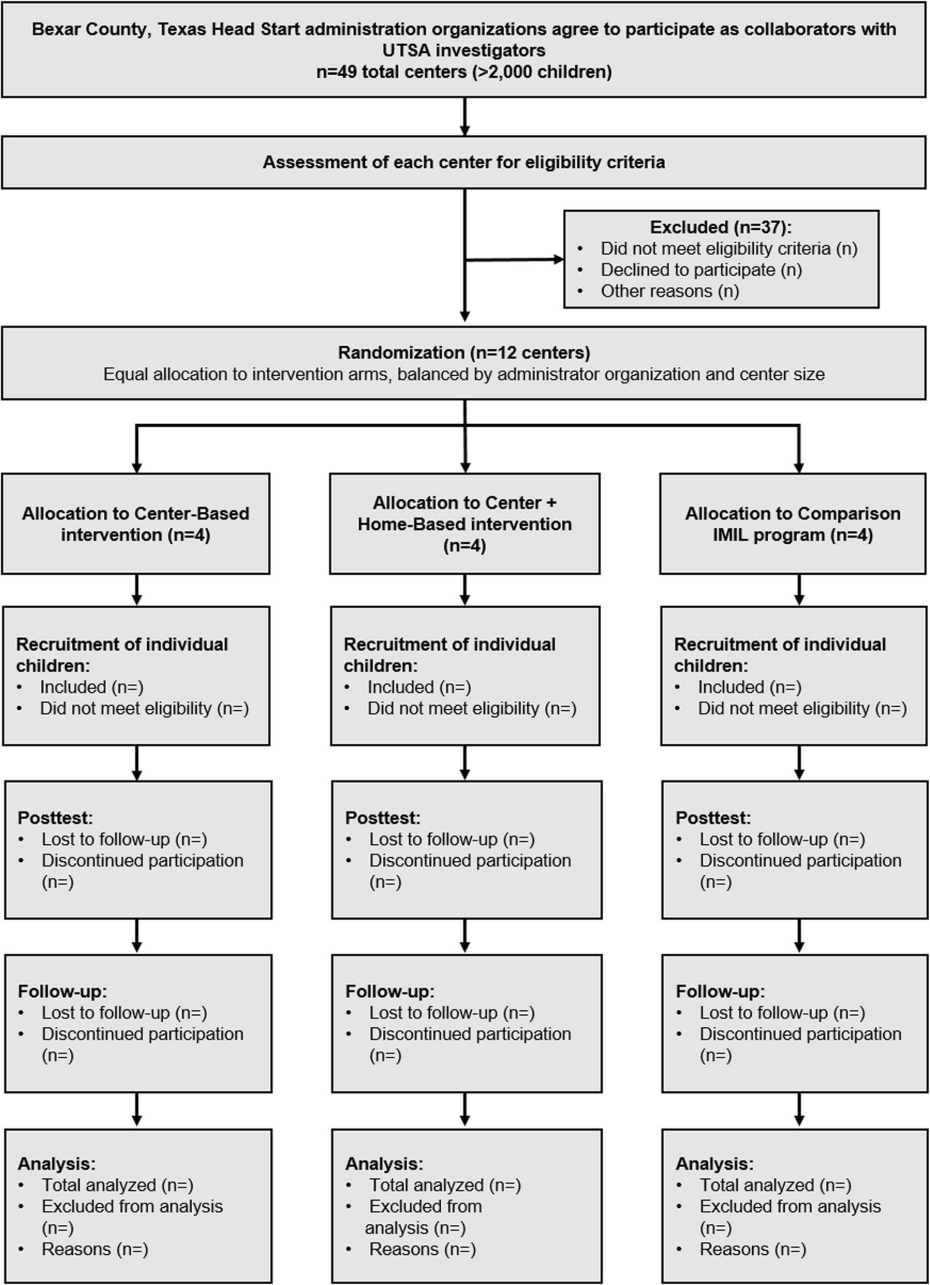
Study Participant Flow

Figure 2 provides an overview of the trial flow for the study.

### Study measures

Data will be collected to assess the primary and secondary study outcomes as well as mediation/moderation measures to evaluate the impacts of the intervention. Table 7 shows the measurements and assessment timelines for the study. We have selected the measures that have established validity and reliability in the study population. All measures for the parents are offered in English and Spanish. To increase parental participation and compliance with assessment protocol, we will provide incentives (up to $30) that are linked to returning daily food/screen time/sleep logs ($3/d) and parent surveys ($9 and raffles for tricycle) at each assessment time. Prior to each data collection, we will provide training on data collection protocols, including privacy protections, to all assessment staff..

**Table 7:** Study measures and assessment timelines

Primary outcomes	Baseline	Posttest	Follow-up
Height, weight, Body Mass Index (BMI; kg/m^2^); BMI z-score [59]	C	C	C
Secondary outcomes			
7-day accelerometry for PA, sedentary time and sleep [60]	C	C	C
The Test of Gross Motor Skills	C	C	C
Aggregated plate waste test^,^ (241)	C	C	C
National Health and Nutrition Examination Survey (NHANES) Dietary Screener			
7-day logs for child screening and sleep time (min/d) [61]	P	P	P
Computer-assisted pictorial test for food preferences [62]	C	C	C
Mediator/moderator measures			
The Home Self-administered Tool for Environmental Assessment of Activity and Diet [63]			
The Parenting Strategies for Eating and Activity Scale [56]	P	P	P
Parent health knowledge test [43]	P	P	P
Parental confidence/self-efficacy scale [64]	P	P	P
Food Behavior Checklist [65]	P	P	P
Acculturation scale [66–68]	P		
Parent weight and physical activity [69]	P	P	P
Family socio-demographics/health history	P		

Data source: *C* child, *P* parent, *S* staff

The primary outcome of the study is child’s BMI calculated as weight in kilograms divided by height in meters squared. Child’s height and weight will be measured twice at the beginning of the school day with no shoes and light clothes, using a stadiometer and digital weight scale. Discrepancy between the two measures must be ≤0.5 cm and ≤ 0.25 kg. We will measure every fifth child by two staff to assure the accuracy and reliability of the weight and height measures.

BMI, BMI-percentile, and zBMI for age and gender will be calculated using the average of the two measures-based child growth charts [59]. We chose change in BMI as the primary endpoint because BMI is within-child referenced [70] and more suitable for assessing change in adiposity in intervention studies of same-age children during the adiposity rebound period [71], compared to zBMI and BMI-percentile. BMI also correlates better with directly measured adiposity in young children [71, 72]. We will analyze zBMI as an outcome measure as well [73]. The selection of the measures for the secondary outcomes and mediation/moderation effects are intended to examine the pathways of influences of policy and behavior changes in the primary outcome depicted in the conceptual model of ¡*Míranos!* intervention. Previous research has shown that these measures play key roles in influencing the levels of obesity in children who participate in lifestyle intervention studies.

### Process evaluation

*The design of* process evaluation is informed by the NIH Behavior Change Consortium Treatment Fidelity Workgroup’s best practice recommendations [74] and recent multi-component RCTs [75]. Because *¡Míranos!* has multiple components with multiple activities, we will use multiple indicators to evaluate the fidelity and completeness of the implementation of all components and determine contribution of each component by linking it to the primary and secondary outcomes. We will collect both quantitative and qualitative data to assess cross-site treatment consistence and non-treatment-related effects [74] and document protocol changes [75]. The evaluation will target three aspects of intervention implementation.

First, the intervention dose delivered (i.e., the extent to which the intervention is delivered as planned to Head Start staff, children and parents) will be measured by: 1) pre- and post-study center environmental assessment by the Environment and Policy Assessment and Observations (EPAO) and auditing of meal menus; 2) completion of delivery schedule of staff and peer leader training, parent education sessions and home visits; 3) evaluation of staff and peer leader training; 4) monthly auditing of weekly lesson plans; and 5) monthly checklist of use of *Míranos!* Activity Cards, children’s story books, and HHL learning activities.

Second, the intervention dose received (i.e., the extent to which Head Start children and parents understand and learn the knowledge and skills delivered in the intervention by Head Start staff) will be assessed by: 1) attendance records of staff and peer leader training and parent education sessions; 2) certification test of staff and peer leader training; 3) monthly staff evaluation (rating scale) of children’s learning of HHL content, gross motor skills and eating behaviors; 4) post-study parent intercept interviews; and 5) post-study focus groups on program delivery process with staff (*n* = 24) and parents (*n* = 32).

Third, participants’ responses to the intervention (i.e., the extent to which Head Start staff, children and parents use and apply the knowledge and skills learned in the intervention in daily life) will be evaluated by: 1) quarterly observation of staff behavior (rating scale) during outdoor play and lunch by research staff; 2) quarterly assessment on children’s PA by the System for Observing Fitness Instruction Time for Preschoolers [76] and diet by group plate waste test [77] in three randomly selected centers; 3) pre- and post-study home environmental changes by HomeSTEAD [63] and the use of *Míranos!* Action Plan by the parents; 4) in-depth interviews with staff (*n* = 48) for program feedback; and 5) post-study staff and parent evaluation (rating scale) of satisfaction with intervention components/activities.

### Statistical analysis

#### Power and sample size

Our pilots had only modest effect on BMI because of short duration and limited policy changes. As a result, we based our sample size on the effect size (δ) of cohort 1 of Hip Hop to Health Jr. Study (HHHJr.), which had a similar CBI (without center policy changes) and a newsletter-based HBI [73, 78]. In comparison, our proposed CBI and HBI are longer and more intensive. At 1 year post intervention of the HHHJr. study, mean BMI decreased in the intervention group (baseline: Mean = 0.05, SE = 0.05; follow-up: Mean = 0.02, SE = 0.11) and increased in the control group (baseline: Mean = 0.14, SE = 0.05; follow-up: Mean = 0.64, SE = 0.11) with a mean group difference of − 0.53 (mean change of − 0.03 in the intervention and 0.50 in the control group) and intraclass correlation (ρ) of 0.003. With an average SD of 1.147 (1.153 in intervention and 1.141 in control), the δ was − 0.53/1.147 = 0.462. Similar δ and ρ remained at Year 2 follow-up. Similar δ was also reported in successful community- and primary care-based based pilot studies in preschool Latino children [79, 80]. For this study, a sample size of 12 centers (i.e., 4 centers/group) with 29 children per center will achieve 80% power to detect a group difference (i.e., CBI and C&HBI vs. control) of 0.53 BMI units at T1 and T2 using a two-sided t-test with a significance level of 5%, assuming ρ = 0.003 and SD = 1.147 (PASS Version 11, NCSS Kaysville Utah 2011). The final sample size was increased to 37 (> 29/0.8) children per center (i.e., 37 × 12 = 444 children in total) to account for an attrition rate of 80%.

#### Data analysis plan

For Hypothesis 1, we will first calculate the change in BMI at T1 from T0 and at T2 from T0 for each child. We will then compare the difference in change scores among the three intervention groups using ANOVA test or Kruskal-Wallis test at each time point separately. Multiple comparisons defined by various linear combinations of groups (i.e., CBI-control; C&HBI-control) will be performed to exam the difference in change scores with Bonferroni adjustment. To utilize all three measures from each child, generalized linear mixed effects models (GLMMs) will be used to examine the group differences at different time points with BMI as the response variable, and time (3 levels: T0, T1 and T2) and group (3 levels) and their interaction as the explanatory variables. Two random effects will be included in a GLMM: one to account for the correlation among children nested within the same center, and one to account for the three repeated measures of each outcome within the same child. Center size and cohort, as fixed effects, will be included in the model as well. We will also include baseline BMI, child gender and race/ethnicity, and other covariates (e.g., parent characteristics and health behaviors) in the model as needed. Model fit will be assessed by residual diagnostics [81] to guide the best fit model. For Hypothesis 2, we will use the same statistical procedure for testing Hypothesis 1 to analyze the secondary outcome measures (i.e., MVPA, sedentary behavior, TV watching, sleep, and dietary measures), with baseline BMI as a covariate as well to check for differential effect of intervention associated with children’s level of adiposity. When there are missing data, we will compare the dropouts and completers on demographics and various outcome measures using available data. If data are missing at random (MAR) [82], standard computational algorithms such as EM implemented in statistical software allow the use of all the data available to generate appropriate parameter estimates. If the MAR assumption is in doubt, we will conduct sensitive analysis to impute missing data using the multiple imputation with chained equations approach [83]. Specifically, we will impute missing values by adjusting for time of measurement and demographics to create 10 imputed datasets. We will then combine the effect sizes using the Rubin’s rules [84]. Stata/SE (version 15, College Station, Texas) or SAS (version 9.3, Cary, North Carolina) will be used for conducting all analyses proposed.

#### Mediation analysis

We will test for mediation effects following the 4-step procedures outlined by MacKinnon et al. [85] to investigate the behavioral pathways between the intervention and outcomes as depicted in Fig. 1 (i.e., if the intervention worked as designed) using structural equation models [86]. A series of GLMMs will be conducted to examine if the strength of the association between the intervention and each outcome of interest is modified when controlling for each mediator. Specifically, we will test the direct and indirect effect of intervention on study outcomes (i.e., child BMI and secondary outcome measures) through each mediator (i.e., intra- and inter-personal and environmental variables) adjusting for covariates (e.g., demographics and baseline measure) as appropriate.

#### Cost-effectiveness (CE) analysis

We will conduct a trial-based analysis to estimate the CE of *¡Míranos!* compared to no intervention, following standard methods for economic evaluation [87]. Three treatment approaches will be compared based on direct observation of the impact of *¡Míranos!* on cost and effect (i.e., BMI): 1) CBI + HBI; 2) CBI; and 3) control. The economic evaluation will be conducted from the perspective of the program provider. We will use the ingredient approach to estimate the program delivery costs incurred in implementing *¡Míranos!*, which multiplies units of resource utilization with unit costs [88]. Resource utilization and unit costs for the program perspective will be measured by collecting information on: 1) food preparation records and PA equipment; 2) delivery of classroom activities; 3) delivery of HBI; and 4) staff training including peer parent educators and monetary incentives per school. Net intervention costs will be calculated by subtracting usual program costs in absence of the intervention from the intervention costs. All costs will be discounted at 3% per annum where applicable and will be expressed in 2018 U.S. dollars. The CE will be calculated based on mean BMI reduction for each approach using the individual-level data. The cost and health outcome of each approach will be synthesized to calculate incremental CE ratios (in terms of net intervention cost per unit BMI reduction) compared to the next effective approach. Any dominated or weakly-dominated strategy will be excluded. Sampling (or stochastic) uncertainty inherent in the trial-based economic evaluation will be evaluated using cost-effectiveness acceptability curves (CEACs), which estimate the probability that an intervention would be cost-effective under varying ranges of willingness-to-pay thresholds for a unit reduction in BMI. CEACs will be constructed using nonparametric bootstrapping with 2000 replicates [87]. STATA 14 for Windows (StataCorp LP, College Station, Texas) or SAS (version 9.3, Cary, North Carolina) will be used for conducting the bootstrap analyses.

### Plan for dissemination

In addition to publications and professional presentations on study outcomes, we plan to create a dissemination package of the *¡Míranos!* intervention that includes: 1) manuals describing intervention components; 2) procedures and resources for implementation and evaluation; 3) fixed costs, variable costs, and CE associated with the intervention; 4) staff training modules; and 5) the intervention’s feasibility and acceptability to inform others in their decisions for adoption [89]. We also plan to work with the National Head Start Association, YMCA, and other childcare organizations to facilitate the translation of *¡Míranos!* into real-world settings if it is shown to be efficacious in the proposed study.

### Data management plan

Study data will be stored in a secured database. All data collection forms will be processed and stored in a secured location. Research staff will enter collected data into the database. Data collection forms will be reviewed immediately after collection for missing or ambiguous information so that clarification or corrections can be made promptly. Data entry quality control will be performed following the double data entry procedure. Quarterly quality control reports will be reviewed and remediation (e.g., re-training) will occur promptly as needed.

### Data safety and monitoring

Because this study is a behavioral intervention of minimal risk, the data safety and monitoring will be performed by an independent monitor. Participants accrual (adherence to protocol regarding demographics, inclusion/exclusion) and retention will be reported at the end of each data collection wave. Compliance to intervention protocol and adverse event rates will be reviewed quarterly. The stopping rules that might be relevant would be: 1) study recruitment or retention becomes futile; or 2) any new information concerning PA and dietary recommendations or safety becomes available during the trial that necessitates stopping the trial. We have no plan to perform interim data analysis.

## Discussion

To our knowledge, the *¡Míranos!* intervention is the first preschool obesity prevention study using an integrated approach to address multiple EBRBs in multiple settings in low-income young children in the United States. The design of the study is informed by recent childhood obesity prevention interventions with considerations of programming and methodological issues identified in the literature [90, 91]. Many of these studies, however, were conducted in Australia and European countries. This study will provide much needed information on the cost-effectiveness of an obesity prevention program in a U.S. child care setting.

The design of this study has several important strengths and weaknesses that may influence the validity and generalizability of the study findings. First, this is a clustered RCT with a long-term follow-up assessment that is made possible by the commitment and support of two Head Start organizations. Findings from this study will provide answers to many questions on the impacts of a multi-faceted intervention targeting both the childcare and home environment. Second, *¡Míranos!* CBI will implement a comprehensive set of center policies and staff training that will address many of the enablers and barriers in a childcare setting. The feasibility and acceptability of these evidence-based policies will be critically examined by the process evaluation data. Previous RCT studies have not provided detailed evaluations of these critical issues in a childcare setting. Third, the intervention activities are tailored to address the barriers facing children and families living in low-income and predominantly Latino communities. A lack of cultural tailoring has been identified as a limitation in previous studies. Finally, *¡Míranos!* intervention is grounded in social cognitive and behavioral theories. This study will test the influences of mediators and moderators on the study outcomes based on the intervention model.

The limitations of the study include the problem of concealment. The Head Start staff are not blinded to the treatment assignment. Concealment of the assignment is not possible because of the need to plan intervention logistics with the administrative staff and conduct the staff training before the start of the school year. It is not clear how staff participation will be biased by this practice. We purposefully enhanced the program for the comparison centers to include the provision of the IMIL program and a parent literacy education program. We hope that the use of an active control program will increase the appeal to the comparison participants and retain them in the study. Another limitation is that the data collection staff will also not be blinded to the treatment assignment. This is especially true for the assessment of the second cohort participants since all of the intervention centers will have many visible signs of *¡Míranos!* intervention. We will address this weakness by conducting a standardized protocol of assessment and close monitoring of the measurement of the primary outcome.

Early childhood obesity is a complex health problem especially among low-income and minority children. There is currently limited evidence on effective prevention strategies based on RCTs in this age group. Designed as an efficacy study, the *¡Míranos!* intervention has been tailored for low-income, Latino preschool children and parents following recent recommendations and guidelines for obesity prevention targeting childcare and home environments. As such, the proposed study can contribute to the evidence base on this important public health concern.

## Additional file

Additional file 1: Samples of Activity Cards. (PDF 242 kb)

## Abbreviations

¡*Míranos*: ¡*Míranos*! Look at Us, We Are Healthy!
BMI: Body Mass Index
BMI-percentile: BMI-percentile for age and gender
CACFP: The Child and Adult Care Food Program
CBI: Center-based intervention
CE: Cost-effectiveness
CEACs: Cost-effectiveness acceptability curves
EBRBs: Energy-balance-related behaviors
GLMMs: Generalized linear mixed effects models
HBI: Home-based intervention
HHL: The Sesame Workshop bilingual Healthy Habits for Life
IMIL: I am moving, I am learning
MAR: Missing at random
MVPA: Moderate to vigorous physical activity
PA: Physical activity
RCT: Randomized controlled trial
T0: Baseline
T1: Immediate post-intervention
T2: 1-year post-intervention follow-up
U.S.: The United States
UTSA: The University of Texas at San Antonio
zBMI: BMI z-scores for age and gender

## References

[1] Guo SS (2002). Predicting overweight and obesity in adulthood from body mass index values in childhood and adolescence. Am J Clin Nutr.

[2] Dietz WH (1998). Health consequences of obesity in youth: childhood predictors of adult disease. Pediatrics.

[3] Schwartz MB, Puhl R (2003). Childhood obesity: a societal problem to solve. Obes Rev.

[4] Kosti RI, Panagiotakos DB (2006). The epidemic of obesity in children and adolescents in the world. Cent Eur J Public Health.

[5] Ogden CL, Carroll MD, Kit B, Flegal KM (2014). Prevalence of childhood and adult obesity in the United States, 2011-2012. JAMA.

[6] Ogden CL, Lamb MM, Carroll MD, Flegal, KM. Obesity and socioeconomic status in children: United States, 2005–2008. NCHS data brief no 51. Hyattsville: National Center for Health Statistics; 2010.

[7] Singh GK, Siahpush M, Kogan MD (2010). Rising social inequalities in US childhood obesity, 2003–2007. Ann Epidemiol.

[8] Wang Y, Zhang Q (2006). Are American children and adolescents of low socioeconomic status at increased risk of obesity? Changes in the association between overweight and family income between 1971 and 2002. Am J Clin Nutr.

[9] Centers for Disease Control and Prevention (2013). Obesity prevalence among low-income, preschool-aged children--new York City and Los Angeles County, 2003-2011. MMWR Morb Mortal Wkly Rep.

[10] De Craemer M, De Decker E, De Bourdeaudhuij I, Vereecken C, Deforche B, Manios Y (2012). Correlates of energy balance-related behaviours in preschool children: a systematic review. Obes Rev.

[11] Hill JO (2006). Understanding and addressing the epidemic of obesity: an energy balance perspective. Endocr Rev.

[12] Swinburn B, Caterson I, Seidell J, James W (2004). Diet, nutrition and the prevention of excess weight gain and obesity. Public Health Nutr.

[13] Wang YC, Orleans CT, Gortmaker SL (2012). Reaching the healthy people goals for reducing childhood obesity: closing the energy gap. Am J Prev Med.

[14] Reilly JJ (2008). Physical activity, sedentary behaviour and energy balance in the preschool child: opportunities for early obesity prevention. Proc Nutr Soc.

[15] LeBlanc AG, Spence JC, Carson V, Connor Gorber S, Dillman C, Janssen I (2012). Systematic review of sedentary behaviour and health indicators in the early years (aged 0–4 years). Appl Physiol Nutr Metab.

[16] St-Onge MP (2013). The role of sleep duration in the regulation of energy balance: effects on energy intakes and expenditure. J Clin Sleep Med.

[17] O’Connor TM, Yang S-J, Nicklas TA (2006). Beverage intake among preschool children and its effect on weight status. Pediatrics.

[18] American Academy of Pediatrics APHA, National Resource Center for Health and Safety in Child Care and Early Education. Caring for our children: National health and safety performance standards; Guidelines for early care and education programs Elk Grove Village, IL. 3rd ed. Washington, DC: American Academy of Pediatrics, American Public Health Association; 2011. [cited 2012 September] Available from: https://nrckids.org/files/CFOC3_updated_final.pdf.

[19] National Association for Sport and Physical Education. Active Start: A Statement of Physical Activity Guidelines for Children From Birth to Age 5 Reston (VA). 2nd ed: NASPE Publishers; 2009. Available from: https://www.shapeamerica.org/standards/guidelines/activestart.aspx.

[20] Story M, Kaphingst K, French S (2006). The role of child care settings in obesity prevention. Future Child.

[21] Trost SG, Sirard JR, Dowda M, Pfeiffer KA, Pate RR (2003). Physical activity in overweight and nonoverweight preschool children. Int J Obes Relat Metab Disord.

[22] Morano M, Colella D, Caroli M (2011). Gross motor skill performance in a sample of overweight and non-overweight preschool children. Int J Pediatr Obes.

[23] Bornstein DB, Beets MW, Byun W, McIver K (2011). Accelerometer-derived physical activity levels of preschoolers: a meta-analysis. J Sci Med Sport.

[24] Vale S, Silva P, Santos R, Soares-Miranda L, Mota J (2010). Compliance with physical activity guidelines in preschool children. J Sports Sci.

[25] Anderson SE, Whitaker RC (2010). Household routines and obesity in US preschool-aged children. Pediatrics.

[26] Seegers V, Petit D, Falissard B, Vitaro F, Tremblay RE, Montplaisir J (2011). Short sleep duration and body mass index: a prospective longitudinal study in preadolescence. Am J Epidemiol.

[27] Snell EK, Adam EK, Duncan GJ (2007). Sleep and the body mass index and overweight status of children and adolescents. Child Dev.

[28] Must A, Parisi SM (2009). Sedentary behavior and sleep: paradoxical effects in association with childhood obesity. Int J Obes.

[29] Summerbell CD, Moore HJ, Vögele C, Kreichauf S, Wildgruber A, Manios Y (2012). Evidence-based recommendations for the development of obesity prevention programs targeted at preschool children. Obes Rev.

[30] Krebs-Smith SM, Guenther PM, Subar AF, Kirkpatrick SI, Dodd KW (2010). Americans do not meet federal dietary recommendations. J Nutr.

[31] Banfield EC, Liu Y, Davis JS, Chang S, Frazier-Wood AC (2016). Poor adherence to US dietary guidelines for children and adolescents in the National Health and nutrition examination survey population. J Acad Nutr Diet.

[32] National Cancer Institute. Usual dietary intakes: food intakes, U.S. population, 2007–10: epidemiology research program web site, 2015. Available from: https://epi.grants.cancer.gov/diet/usualintakes/pop/2007-10/.

[33] Birch LL, Davison KK (2001). Family environmental factors influencing the developing behavioral controls of food intake and childhood overweight. Pediatr Clin N Am.

[34] Birch LL, Fisher JO (1998). Development of eating behaviors among children and adolescents. Pediatrics.

[35] Dubois L, Farmer AP, Girard M, Peterson K (2006). Preschool children's eating behaviours are related to dietary adequacy and body weight. Eur J Clin Nutr.

[36] Institute of Medicine (2011). Early childhood obesity prevention policies.

[37] Birch LL, Johnson SL, Andresen G, Peters JC, Schulte MC (1991). The variability of young Children's energy intake. N Engl J Med.

[38] Huang TT, Drewnosksi A, Kumanyika S, Glass TA (2009). A systems-oriented multilevel framework for addressing obesity in the 21st century. Prev Chronic Dis.

[39] Kremers S, de Bruijn G-J, Visscher T, van Mechelen W, de Vries N, Brug J (2006). Environmental influences on energy balance-related behaviors: a dual-process view. Int J Behav Nutr Phys Act.

[40] Ward DS, Vaughn A, Story M (2013). Expert and stakeholder consensus on priorities for obesity prevention research in early care and education settings. Child Obes.

[41] Kaphingst KM, Story M. Child care as an untapped setting for obesity prevention: state child care licensing regulations related to nutrition, physical activity, and media use for preschool-aged children in the United States. Prev Chronic Dis. 2009;6(1):A11. PMC2644584 19080017

[42] O’Brien M, Nader PR, Houts RM, Bradley R, Friedman SL, Belsky J (2007). The ecology of childhood overweight: a 12-year longitudinal analysis. Int J Obes.

[43] Yin Z, Parra-Medina D, Cordova A, He M, Trummer V, Sosa E (2012). Miranos! Look at us, we are healthy! An environmental approach to early childhood obesity prevention. Child Obes.

[44] He M, Sosa E, Cordova A, Wilmoth S, Bustos D, Perez A (2015). Effects of healthy eating promotion on food preference of head start preschoolers. J Res Obes.

[45] Sosa ET, Parra-Medina D, He M, Trummer V, Yin Z. ¡Miranos! (Look at Us! We Are Healthy!): home-based and parent peer–led childhood obesity prevention. Health Promot Pract. 2016;17(5):675–81. 10.1177/1524839915623762 26895848

[46] 110th Congress. “Improving Head Start for School Readiness Act of 2007” Public Law 110–134 (2007).

[47] Tarullo LWJ, Aikens N, Hulsey L (2008). Beginning head start: children, families, and programs in fall 2006.

[48] US Department of Health and Human Services Administration for Children and Families Head Start Bureau. Office of Head Start: legislation & regulations2012; 2012 (September 9). Available from: https://www.acf.hhs.gov/ohs/policy.

[49] Hughes CC (2010). Barriers to obesity prevention in head start. Health Aff.

[50] Whitaker RC (2009). A national survey of obesity prevention practices in head start. Arch Pediatr Adolesc Med.

[51] Wang Y, Gortmaker S, Sobol A, Kuntz K (2006). Estimating the energy gap among US children: a counterfactual approach. Pediatrics.

[52] Sacks G, Swinburn B, Lawrence M (2009). Obesity policy action framework and analysis grids for a comprehensive policy approach to reducing obesity. Obes Rev.

[53] Gortmaker SL, Swinburn BA, Levy D, Carter R, Mabry PL, Finegood DT (2011). Changing the future of obesity: science, policy, and action. Lancet.

[54] Swinburn B, Egger G (2002). Preventive strategies against weight gain and obesity. Obes Rev.

[55] Birch LLFJO (1998). Development of eating behaviors among children and. Pediatrics.

[56] Larios SE, Ayala GX, Arredondo EM, Baquero B, Elder JP (2009). Development and validation of a scale to measure Latino parenting strategies related to children’s obesigenic behaviors: the parenting strategies for eating and activity scale (PEAS). Appetite.

[57] Bahn D (2001). Social learning theory: its application in the context of nurse education. Nurse Educ Today.

[58] Dwyer GM, Higgs J, Hardy LL, Baur LA (2008). What do parents and preschool staff tell us about young children's physical activity: a qualitative study. Int J Behav Nutr Phys Act.

[59] Centers for Disease Control and Prevention. CDC Growth Charts: United States 2000 [updated June 12, 2002. Available from: https://www.cdc.gov/growthcharts/clinical_charts.htm.

[60] Addy CL, Trilk JL, Dowda M, Byun W, Pate RR (2014). Assessing preschool children's physical activity: how many days of accelerometry measurement. Pediatr Exerc Sci.

[61] Haines J, McDonald J, O’Brien A (2013). Healthy habits, happy homes: randomized trial to improve household routines for obesity prevention among preschool-aged children. JAMA Pediatr.

[62] Jaramillo SJ, Yang S-J, Hughes SO, Fisher JO, Morales M, Nicklas TA (2006). Interactive computerized fruit and vegetable preference measure for African-American and Hispanic preschoolers. J Nutr Educ Behav.

[63] Hales D, Vaughn A, Mazzucca S, Bryant M, Tabak R, McWilliams C (2013). Development of HomeSTEAD's physical activity and screen time physical environment inventory. Int J Behav Nutr Phys Act.

[64] Taveras EM, Mitchell K, Gortmaker SL (2009). Parental confidence in making overweight-related behavior changes. Pediatrics.

[65] Townsend MS, Kaiser LL, Allen LH, Block Joy A, Murphy SP (2003). Selecting items for a food behavior checklist for a limited-resource audience. J Nutr Educ Behav.

[66] Marin G, Gamba RJ (1996). A new measurement of acculturation for Hispanics: the Bidimensional acculturation scale for Hispanics (BAS). Hisp J Behav Sci.

[67] Power TG, O’Connor TM, Orlet Fisher J, Hughes SO (2015). Obesity risk in children: the role of acculturation in the feeding practices and styles of low-income Hispanic families. Child Obes.

[68] Ayala GX, Baquero B, Klinger S (2008). A systematic review of the relationship between acculturation and diet among Latinos in the United States: implications for future research. J Am Diet Assoc.

[69] Centers for Disease Control and Prevention (CDC). Behavioral Risk Factor Surveillance System Survey Questionnaire Atlanta, Georgia 2011 [Available from: https://www.cdc.gov/brfss/questionnaires/pdf-ques/2011brfss.pdf.

[70] Cole TJ, Faith MS, Pietrobelli A, Heo M (2005). What is the best measure of adiposity change in growing children: BMI, BMI %, BMI z-score or BMI centile?. Eur J Clin Nutr.

[71] Field AE, Laird N, Steinberg E, Fallon E, Semega-Janneh M, Yanovski JA (2003). Which metric of relative weight best captures body fatness in children?. Obes Res.

[72] Kakinami L, Henderson M, Chiolero A, Cole TJ, Paradis G (2014). Identifying the best body mass index metric to assess adiposity change in children. Arch Dis Child.

[73] Fitzgibbon ML, Stolley MR, Schiffer L, Van Horn L, KauferChristoffel K, Dyer A (2005). Two-year follow-up results for hip-hop to health Jr.: a randomized controlled trial for overweight prevention in preschool minority children. J Pediatr.

[74] Bellg AJ, Borrelli B, Resnick B, Hecht J, Minicucci DS, Ory M (2004). Enhancing treatment Fidelity in health behavior change studies: best practices and recommendations from the NIH behavior change consortium. Health Psychol.

[75] Pfeiffer K, Saunders R, Brown W, Dowda M, Addy C, Pate R (2013). Study of health and activity in preschool environments (SHAPES): study protocol for a randomized trial evaluating a multi-component physical activity intervention in preschool children. BMC Public Health.

[76] Sharma SV, Chuang R-J, Skala K, Atteberry H (2011). Measuring physical activity in preschoolers: reliability and validity of the system for observing fitness instruction time for preschoolers (SOFIT-P). Meas Phys Educ Exerc Sci.

[77] Jacko C, Deliava J, Ensle K, Hoffman DJ (2007). Use of the plate-waste method to measure food intake in children. J Ext.

[78] Fitzgibbon ML, Stolley MR, Dyer AR, VanHorn L, KauferChristoffel K (2002). A community-based obesity prevention program for minority children: rationale and study design for hip-hop to health Jr. Prev Med.

[79] Laws R, Campbell K, van der Pligt P, Russell G, Ball K, Lynch J (2014). The impact of interventions to prevent obesity or improve obesity related behaviours in children (0–5 years) from socioeconomically disadvantaged and/or indigenous families: a systematic review. BMC Public Health.

[80] Skouteris H, McCabe M, Swinburn B, Newgreen V, Sacher P, Chadwick P (2011). Parental influence and obesity prevention in pre-schoolers: a systematic review of interventions. Obes Rev.

[81] Diggle PJ, Liang KY, Zeger SL (1994). Analysis of longitudinal data.

[82] Little RJA, Rubin DB (2002). Statistical analysis with missing data.

[83] Raghunathan T, Lepkowski JM, Van Hoewyk J, PA S (2001). Multivariate technique for multiply imputing missing values using a sequence of regression models. Surv Methodol.

[84] Rubin DB (1987). Multiple imputation for nonresponse in surveys.

[85] MacKinnon DP, Fairchild AJ, Fritz MS (2007). Mediation analysis. Annu Rev Psychol.

[86] Baranowski T, Klesges LM, Cullen KW, Himes JH (2004). Measurement of outcomes, mediators, and moderators in behavioral obesity prevention research. Prev Med.

[87] Centers for Disease Control and Prevention U.S. Department of Health & Human Services. Cost Analysis [Available from: https://www.cdc.gov/policy/polaris/economics/program-cost.html.

[88] Wolfenstetter SB, Wenig CM (2011). Costing of physical activity programmes in primary prevention: a review of the literature. Health Econ Rev.

[89] Glasgow RE, Emmons KM (2007). How can we increase translation of research into practice? Types of evidence needed. Annu Rev Public Health.

[90] Wolfenden L, Jones J, Williams CM, Finch M, Wyse RJ, Kingsland M (2016). Strategies to improve the implementation of healthy eating, physical activity and obesity prevention policies, practices or programmes within childcare services. Cochrane Database Syst Rev.

[91] Ling J, Robbins LB, Wen F (2016). Interventions to prevent and manage overweight or obesity in preschool children: a systematic review. Int J Nurs Stud.

