# Supplementary material for: Study protocol for a cluster randomized controlled trial to test “¡Míranos! Look at Us, We Are Healthy!” – an early childhood obesity prevention program
Source: BMC Pediatr. 2019 Jun 10;19:190. doi: 10.1186/s12887-019-1541-4 (PMC6556954; doi:10.1186/s12887-019-1541-4)
Supplement: Supplementary file 1 — Samples of Activity Cards. (PDF 242 kb) [file 12887_2019_1541_MOESM1_ESM.pdf]

Builders and Bulldozers

Everyday 5 minutes

Level: Moderate to Vigorous

Skill: Manipulative

Equipment: 12-14 small cones

Activity: Divide the students into 2 groups.
One group will be the builders and one will be the bulldozers.
On the signal to start, the builders will move around the playing area attempting to set up any cones that have been knocked over. The bulldozers will move around the playing area attempting to knock down any cones that are upright. Bulldozers may only use their hands (no kicking the cones) to knock down the cones.
After a few minutes, stop and switch the roles of the students.
Those students who were builders will become bulldozers and bulldozers will become builders for round two.

Modifications:
None, game may be played inside or outside.

Clean Your House

Everyday 5 minutes

Level: Moderate to Vigorous
Skill: Throwing and Retrieving
Equipment: fluff balls (or yarn balls) and cones to mark room division
Activity: Divide your class into two teams and have one team on each side of the room. Scatter fluff balls evenly on each side of the room to start. On the signal, “Clean Your House”, students throw the fluff balls to the other side of the room. Students should not cross onto the other side of the room at any time to retrieve or throw a ball. At the end of the playing time the winning team is the team with the least amount of balls on their side.

Modifications:
Best played inside, may be played outside – substitute beanbags for fluff balls
